# Orthonome – a new pipeline for predicting high quality orthologue gene sets applicable to complete and draft genomes

**DOI:** 10.1186/s12864-017-4079-6

**Published:** 2017-08-31

**Authors:** Rahul V. Rane, John G. Oakeshott, Thu Nguyen, Ary A. Hoffmann, Siu F. Lee

**Affiliations:** 1 Bio21 Institute, School of Biosciences, The University of Melbourne, Melbourne, Victoria Australia; 2 CSIRO, Canberra, Australian Capital Territory Australia; 3 Department of Biological Sciences, Macquarie University, Sydney, New South Wales Australia

**Keywords:** Orthologue, Inparalogue, Gene duplication, Gene birth

## Abstract

**Background:**

Distinguishing orthologous and paralogous relationships between genes across multiple species is essential for comparative genomic analyses. Various computational approaches have been developed to resolve these evolutionary relationships, but strong trade-offs between precision and recall of orthologue prediction remains an ongoing challenge.

**Results:**

Here we present *Orthonome*, an orthologue prediction pipeline, designed to reduce the trade-off between orthologue capture rates (recall) and accuracy of multi-species orthologue prediction. The pipeline compares sequence domains and then forms sequence-similar clusters before using phylogenetic comparisons to identify inparalogues. It then corrects sequence similarity metrics for fragment and gene length bias using a novel scoring metric capturing relationships between full length as well as fragmented genes. The remaining genes are then brought together for the identification of orthologues within a phylogenetic framework. The orthologue predictions are further calibrated along with inparalogues and gene births, using synteny, to identify novel orthologous relationships. We use 12 high quality *Drosophila* genomes to show that, compared to other orthologue prediction pipelines, *Orthonome* provides orthogroups with minimal error but high recall. Furthermore, *Orthonome* is resilient to suboptimal assembly/annotation quality, with the inclusion of draft genomes from eight additional *Drosophila* species still providing >6500 1:1 orthologues across all twenty species while retaining a better combination of accuracy and recall than other pipelines. *Orthonome* is implemented as a searchable database and query tool along with multiple-sequence alignment browsers for all sets of orthologues. The underlying documentation and database are accessible at http://www.orthonome.com.

**Conclusion:**

We demonstrate that *Orthonome* provides a superior combination of orthologue capture rates and accuracy on complete and draft drosophilid genomes when tested alongside previously published pipelines. The study also highlights a greater degree of evolutionary conservation across drosophilid species than earlier thought.

**Electronic supplementary material:**

The online version of this article (10.1186/s12864-017-4079-6) contains supplementary material, which is available to authorized users.

## Background

Distinguishing orthologous and paralogous relationships among gene complements of whole genomes in different eukaryote species is crucial to understanding patterns of functional conservation and change through the course of evolution [1]. Analyses of these patterns could become far more powerful with the rapid growth in the number of species for which whole genome sequences are available.

Several methods have been developed over the past two decades for orthologue prediction, including tree-based methods (Ensembl Compara [2], PANTHER [3] and PhylomeDB [4]), graph-based methods (i.e., based on pairwise comparisons; Best Reciprocal Hits [5], Reciprocal Smallest Distance (RSD), EggNOG [6], Hieranoid [7], InParanoid [8], OMA [9] and OrthoInspector [10]) and meta-methods combining phylogenetic information derived from different databases (e.g. MetaPhors [11]). Most of these methods were developed prior to the widespread availability of whole genome data so they are not well suited to exploit the contextual information (e.g. positional information or splice variation) provided by such data [12]. Additionally, the graph-based methods involve finding genes in pairs of genomes that share the strongest sequence similarity or have the least genetic distance. However, phenomena such as violations of molecular clock behaviour or rapid gene duplications can compromise their performance [1, 13]. Tree-based methods, on the other hand, typically improve the quality of orthologue inference in the presence of such phenomena [13] and further improvements can be made if synteny information is also used (e.g. BBH-LS [14] or the recently developed MSOAR [15–17]). The MSOAR algorithms utilise genomic context in a genome evolution framework to predict orthologues without relying exclusively on either gene homology or conserved gene order [12, 16, 17]. However MSOAR is unsuitable for analysing draft genomes because it requires chromosome-level assignment of annotated gene models. It also uses approximate measures of genetic similarity rather than the more accurate Smith-Waterman alignments.

The Orthonome orthologue prediction pipeline presented here overcomes the issues highlighted above. Orthonome uses whole genome data but is tolerant of fragmented draft genomes. The pipeline also has a toolkit of scripts to enable orthologue-based whole genome phylogenetic analyses. Orthonome delivers high quality orthologue predictions among eukaryote genomes while maintaining high recall compared to other pipelines. However, Orthonome in its current implementation may not be suited to prokaryote genomes where the prevalence of horizontal gene transfers can confound the evolutionary relationships across species [18]. The accuracy and sensitivity of the pipeline are validated using tests developed by Altenhoff*,* et al. [19] and published data for twenty Drosophila genomes, twelve of which have been extensively curated.

## Implementation

### The Orthonome pipeline

**Fig. 1:**
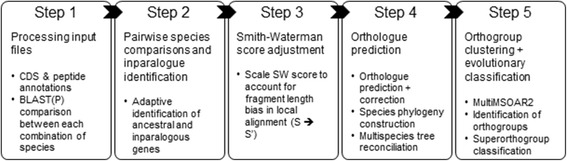
Process flow diagram for the Orthonome pipeline. The five-step pipeline combines the power of heuristic and greedy algorithms to improve the accuracy and recall from both well annotated and draft genomes

Orthonome is a multimodular pipeline and toolkit for orthologue prediction and phylogenetic and gene evolution analysis. The workflow consists of five major steps as summarised in Fig. 1 and detailed as follows:

#### Step 1: Processing input files

Orthonome uses whole genome annotations in GFF3 format and genomes in FASTA format to predict orthology. The processing script ‘Gff2gene_zeroidx.py’ extracts nucleotide coding sequences and their respective translations. This step is tolerant of annotations with gene models split across more than one contig/scaffold, making it applicable to splice-alignment-based gene prediction from fragmented draft assemblies such as those produced by Scipio [1] and ExonMatchSolver-pipeline [2]. Orthonome retains the longest isoform if multiple splice variants are predicted.

Sequence similarity metrics for genes in each species pair are then calculated using BLASTP [3] and Smith-Waterman alignments for each within and between species combination.

#### Step 2: Pair-wise species comparisons and inparalogue identification

Following MSOAR [16], for each pair of species, the BLASTP results from Step 1 are processed to create clusters of genes with similar domains using MCL clustering [20]. Both syntenic and non-syntenic inparalogues are then identified within each cluster using neighbour-joining trees based on DNA distance matrices [21] built using locally optimised codon alignments (MAFFT (L-INS-i) [22] followed by PAL2NAL (v14) [23]). The genes identified as inparalogues are not utilised for further analysis in Step 3, thus retaining only the genes classified as ancestral in origin. This step also serves as a filtering step for genes violating expected phylogenetic relationships, such as horizontal gene transfers (HGT) [18] since they will very likely be identified as spurious inparalogues or *de novo* genes at this stage, depending upon the evolutionary origin of the HGT event in the species analysed.

#### Step 3: Score adjustment for pairwise orthologue prediction

For each gene retained in Step 2, the five genes with highest sequence similarity are identified from the species compared, based on the fragment bias-corrected pairwise gene similarity score, *S*′. These gene pairs are also required to have a low gap extension penalty (−1), Smith-Waterman alignments spanning >50% of matched gene lengths with BLASTP bit scores ≥30, and e-values ≤10e-7. The reduced gap penalty helps accommodate large gaps between coding regions, allowing more efficient recovery of orthologues that have fragmented gene models (Additional file 1: Note 1) or are significantly diverged. The *S*′ scores for each gene pair (G_a1_ and G_a2_) are calculated using a Smith Waterman alignment and BLOSUM50 (BL50) substitution matrix as follows:

$$ {S}^{\prime }={\left(\frac{SmithWaterman^{BL50}\left[{G}_{a1}\  vs\ {G}_{a2}\right]}{Pairwise alignment length\ (bp)}\right)}^{\ast } percent pairwise identity. $$

In the estimation of the *S′* score, the pairwise identity calculated from the alignment is multiplied by 100 to create a percentage metric similar to those used by most other orthologue identification pipelines. The number of most similar genes analysed is restricted to five for the purposes of computing speed, which is unlikely to affect orthologue discovery since most tandem inparalogues have already been removed in Step 2.

#### Step 4: Orthologue prediction and re-calibration to include positional orthologues

Orthonome uses the modified Smith-Waterman scores of the genes and their corresponding genomic coordinates as inputs for the MSOAR algorithm [17] to identify the orthologous relationships between genes of each species pair. Due to the more sensitive identification of orthogonal relationships using the *S′* score, phylogenetically close neighbours including positional inparalogues are re-analysed to re-calibrate orthologue calls after an initial MSOAR run using synteny information from the genome to fill gaps [16] and reassign true orthologues using micro-synteny (spanning windows of six genes). The *S′* scores and syntenic relationships therefore allow Orthonome to extend the MSOAR analysis and recover orthologues misidentified as inparalogues.

The pairwise orthologues are then used to create clusters of genes using the MCL algorithm with an inflation parameter of 2 [20]. FastTree2 is then applied to orthogonal clusters with only one gene from each species, to construct a consensus phylogenetic tree topology based on randomly concatenated codon alignments.

The gene families constructed using an all-by-all matrix of sequence similarity are then reconciled into orthologous clusters by MultiMSOAR2.0 [17]. Tree reconciliation and formation of orthogonal sets of orthologues further utilises the S′ score to quantify sequence similarity between genes. These orthogonal sets of orthologues are termed orthogroups (OG’s).

#### Step 5: Orthogroup clustering and evolutionary classification

Orthonome partitions gene sets into four orthogonal classes, namely orthologues that are present in all species (hereafter termed 1:1 _(n=all)_ orthogroups), those present in some but not all species (1:1 _(1<n<all)_ orthogroups), inparalogues and *de novo* gene births (i.e. no significant sequence homology to any other genes in the data set). The orthogroups and inparalogues are further clustered by domain similarity using protein sequence match followed by repeated MCL clustering with zero edge weights for duplications and *de novo* genes to form super orthologue groups (SOGs). These are akin to functional gene families and consist of orthologues (satisfying at least two of the bi-directional sequence similarity, phylogenetic and syntenic sources of evidence), inparalogues and gene births. Each SOG is named using a four letter alphabet combination code (e.g. ABCD) and orthogroups in the SOG are further numbered as suffixed to the SOG ID (e.g. ABCD-0001, ABCD-0002), while the inparalogues and *de novo* genes are classified with the DUP (inparalogue) and DNV (*de novo*) codes respectively.

The Orthonome pipeline produces tabulated, database-tool-friendly formats which are utilised to build the web interface and an online database (http://www.orthonome.com). The current implementation includes output data from the 20 *Drosophila* species analysed in this study.

#### Computational requirements

The Orthonome pipeline consists of UNIX bash and c++ scripts assisted by toolkits written in Perl and python. Furthermore the pipeline is written in a map-reduce protocol leveraging the parallelisation enabled by a GNU parallel utility [24]. The source code is available at https://bitbucket.org/rahulvrane/orthonome while the database website is hosted at www.orthonome.com.

The pipeline has been tested on Linux servers with four cores and 64 GB memory hosted on the NeCTAR research cloud [25] and requires peak memory usage of 8 GB. The novel map-reduce implementation in Orthonome reduces the time required by the BLASTP search, Smith-Waterman alignment scoring and orthology assessment by 5–10 fold compared to a when programs are run with internal multi-threading, albeit with phylogenetically more distant species still taking longer to compare than more closely related species. The map-reduce based programming models often involve implementing calculations and computational tasks in a parallel distributed manner. The approach to pipeline creation allows optimal usage of computing resources over a computing grid or local computers and is therefore highly scalable from the 20 genomes tested here to hundreds of genomes. An optimised pre-processing step for generation of sequence similarity scores and a 64-bit recompilation of the core algorithms permit completion of pairwise comparative analyses up to three times faster than the original MSOAR2 implementation.

#### Web interface

A complementary web interface is currently available at www.orthonome.com. The web interface allows users to search the database using gene identifiers, gene names or protein/nucleotide sequences in order to view the entire orthogroup and super orthogroup (including genes tagged as inparalogues and closely associated orthogroups clustered by domain) to which the gene in question belongs. Additionally, for each gene analysed in the current implementation, users can also view the summary protein domains [26] and orthologue allocation and interactively view and analyse alignments of the gene with all its orthologues. All the orthologue data and alignments can be downloaded in XLSX, CSV, TXT, FASTA and OrthoXML formats. The output can be directly exported into other data manipulation software.

### Comparisons between Orthonome and other pipelines

#### Data used in the comparisons

The genome and annotation data for the implementation of Orthonome herein use the 12 Flybase *Drosophila* species (*Drosophila ananassae, D. erecta, D. grimshawi, D. melanogaster, D. mojavensis, D. persimilis, D. pseudoobscura, D. sechellia, D. simulans, D. virilis, D. willistoni, D. yakuba*; accessible at ftp://ftp.flybase.net/genomes/) [27] and the additional eight draft genomes in modENCODE (*D. biarmipes, D. bipectinata, D. elegans, D. eugracilis, D. ficusphila, D. kikkawai, D. rhopaloa, D. takahashii*; https://www.hgsc.bcm.edu/arthropods/drosophila-modencode-project) (Additional file 1: Table S1). A total of 308,667 genes were analysed using this approach across the 20 species (Additional file 1: Table S1). We did not use the 66 metazoan species set proposed by Altenhoff*,* et al. [19] because Orthonome depends upon whole genome information, including both genome sequence and annotation, which is unavailable for most of the 66 species.

*The three performance comparisons were carried out as follows:*

1. Comparison between Orthonome and five other orthologue prediction pipelines.

The six orthologue prediction pipelines (Orthonome, OrthoDB [28], Reciprocal bi-directional hit, MetaPhors [11], OMA (two modes: groups and GETHOGS) [9] and MultiMSOAR2 [17]) were compared according to methods which Altenhoff*,* et al. [19] developed to (i) evaluate phylogenetic discordance between species trees and gene trees constructed using orthologue sets, and (ii) measure the number of orthologue clusters with representatives from all species – representing a recall of conserved orthologues. For pipelines that do not produce orthogonal 1:1 orthogroup predictions (e.g. RBH, OrthoDB and GETHOG’s), we used the Quest for Orthologs method (as proposed by the authors) to sample a maximal path across all species, starting with a randomly selected gene and selecting a gene from the ‘next’ species and resolving soft polytomies (or one:many relationships) using random selection and bootstrapping (see Altenhoff*,* et al. [19]). We did not use the gene tree or ontology-based methods of comparison from Altenhoff*,* et al. [19] because only one of the 20 species has the manually annotated gene ontologies and gene trees required for those tests.

2. Comparison between Orthonome and the published data on the 12 drosophila genomes obtained from Flybase.

The output statistics from Orthonome were compared to those generated through the OrthoDB pipeline from Flybase (OrthoDB v7) (https://flybase.org/static_pages/downloads/FB2015_04/genes/gene_orthologs_fb_2015_04.tsv.gz using the script Orthonome_discordance_data.py (see Additional file 1). All orthogroups unique to Orthonome were tested for gene ontology enrichment using the goatools pipeline (https://github.com/tanghaibao/goatools) and enrichment was carried out with reference to the *D. melanogaster* genome.

3. Comparison between Orthonome, OrthoDB and MSOAR2.

We also compared the output of Orthonome to that of OrthoDB and MSOAR2 for 20 *Drosophila* species using the default parameters defined by Kriventseva*,* et al. [28] for OrthoDB and Shi*,* et al. [17] for MSOAR2.

## Results and discussion

### Benchmarking against current pipelines

To assess its performance, we applied Orthonome to datasets of 12 and 20 *Drosophila* species and compared our results to those generated with OrthoDB [29], Reciprocal bi-directional best hit [5], MetaPhors [11], OMA (groups and GETHOGS) [9] and MultiMSOAR2 [16, 17] using two measures described in Altenhoff*,* et al. [19]. Firstly, we calculated the average tree error (Robinson-Foulds distance between the reference species phylogeny and the tree obtained from orthologous clusters with all tested species [19]) for each method using the 12 and 20 genome sets. This test assumes that a true orthologous group with all species represented (1:1 _(n=all)_ orthogroups in the case of Orthonome) should give rise to the same phylogenetic tree as the overall species tree. Therefore a lower average tree error represents better predictive capacity of the pipeline. For the second of the measures we calculated the recall statistic, namely the number of complete (1:1 _(n=all)_ or maximal path search) orthologous group sets recovered by the pipeline, where a higher number indicates better resolution of orthologous relationships.

**Fig. 2:**
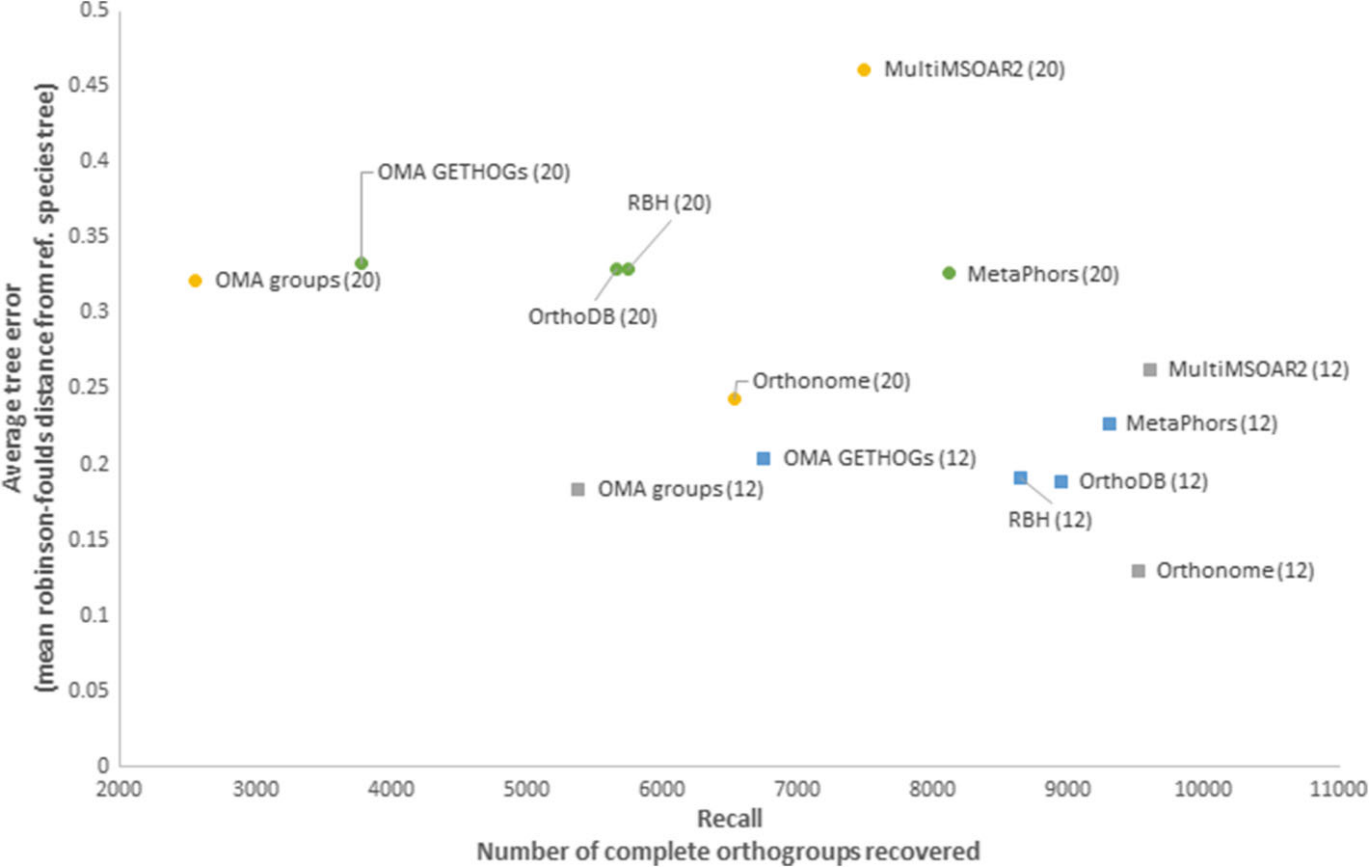
Species tree discordance and orthologue recall tests for six pipelines on the 12 and 20 *Drosophila* species data sets. The circular markers represent the results for the 20 species data set while the square markers represent those for the 12 high quality Flybase genomes. The yellow circular markers and grey square markers represent pipelines that produce orthogonal orthologue sets with only one gene per species in a cluster. Orthonome provides a superior combination of low average tree error and high recall with both data sets

Application of the six pipelines above to the 12 high quality Flybase genomes showed Orthonome had the lowest average tree error rate and second highest recall (9538 1:1 _(n=all)_ orthogroups, Fig. 2). By comparison, MultiMSOAR2, which had the highest recall (9595 1:1 _(n=all)_ orthogroups), also had the highest average tree error rate. The performance of Orthonome is likely to reflect improved pairwise orthologue identification due to use of the *S′* score. The latter uses the more accurate Smith Waterman alignment rather than heuristic BLAST alignments and accounts for weighted similarity per amino acid, which will further prefer the orthologous relationship between species over recently formed inparalogues. OMA (groups), which had the second lowest average tree error rate had nearly four-fold lower recall compared to Orthonome. Additionally, OrthoDB, which is the most widely accepted source of orthologues for the *Drosophila* species [30], also had lower quality (3rd best) and recall (4th best) compared to Orthonome. Therefore, for the 12 genomes we found that Orthonome had the best combination of average tree error rate and recall compared to the six other pipelines tested.

Addition of the eight lower quality modENCODE genomes to the data set yielded a broadly similar pattern of differences among the pipelines [Fig. 2]. The recall rate for Orthonome was now rated 3rd best, but it still had the best combination of recall and accuracy. The lower number of 1:1 _(n=all)_ orthogroups recovered by all pipelines from the 20 genome data set is most likely due to the lower quality annotations (e.g. chimeric and missing genes) in the eight modENCODE genomes [31].

### Detailed comparison of Orthonome with OrthoDB using the 12 genome data set

**Fig. 3:**
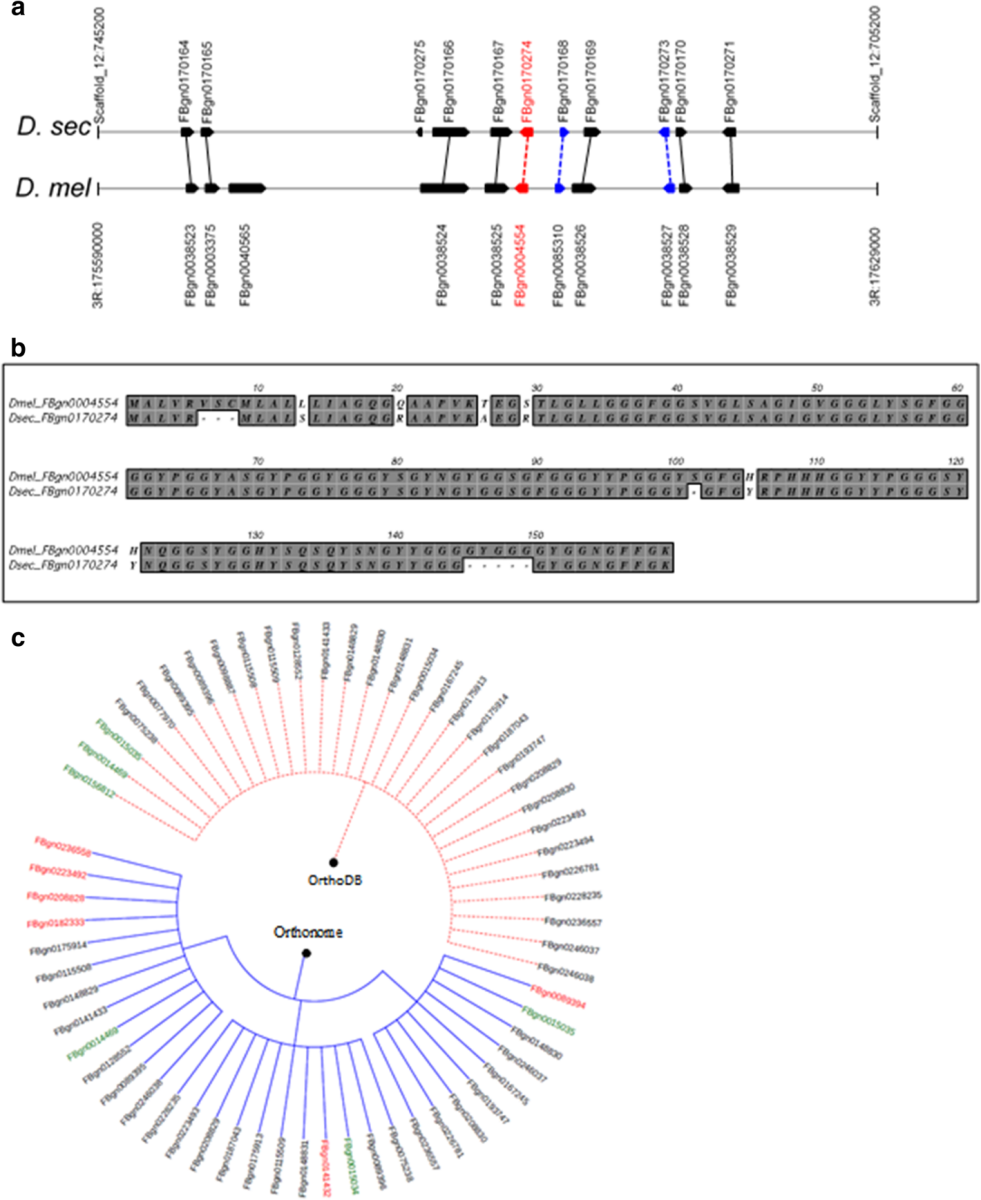
Comparison between Orthonome, MSOAR and OrthoDB for orthologue identification across syntenically supported regions and fast evolving gene families. **a** A comparison of orthologue capture success in a 40 Kb syntenic region between *D. sechellia* and *D. melanogaster*. Orthologous relationships are indicated by vertical lines. Black lines = orthologous pairs supported by OrthoDB, MSOAR and Orthonome; blue lines = orthologous pairs detected only by MSOAR and Orthonome; red line = orthologous pair recovered only by Orthonome. **b** The newly recovered orthologous pair FBgn0004554 and FBgn0170274 in Panel A is supported by high (>90%) amino acid identity. **c** Orthonome is able to split OrthoDB orthogroup EOG7KHP47 (dotted orange clade) consisting of P450 monooxygenases into three independent orthogroups (solid blue clade). The three *D. melanogaster* genes are highlighted in green and the six additional genes that were allocated to the orthogroups by Orthonome only are marked in red. OrthoDB was unable to identify orthology to these six genes

The analysis of the 12 well curated *Drosophila* genomes in Flybase with Orthonome identified orthologous relationships for an average of ~1.8% more genes in each species than did OrthoDB (13,516 vs 13,270), including resolving cryptic relationships between multiple genes and discovering more novel orthologous relationships. One such example is the orthologous pair: *D. melanogaster* gene FBgn0004554 and its *D. sechellia* counterpart FBgn0170274 (see e.g. in Fig. 3a/b and Additional file 1: Note 2). Orthonome also found ~44% more 1:1 _(n=all)_ orthogroups (9538 vs 6621) than did OrthoDB (Table 1, Additional file 1: Table S1 [29]). Specifically, it resolved an additional 2917 1:1 _(n=all)_ orthogroups from among orthogroups that OrthoDB had left as 1:many or many:many sets (Fig. 3c and Additional file 1: Table S2). Furthermore, 94%, or 6228, of the 1:1 _(n=all)_ orthogroups predicted by OrthoDB were also predicted by Orthonome.

**Table 1:** Numbers of orthologues, orthogroups, inparalogues and gene births identified by Orthonome, OrthoDB, MultiMSOAR2, OMA groups and reciprocal best hit (RBH) using the same input data

Comparison	Measures	Orthonome	OrthoDB	Multi MSOAR2	OMA	RBH
Twelve FlyBase genomes	Average number of genes per species	15,446				
	Average number of orthologues per species	13,517	13,270	13,310	12,934	13,545
	Average number of inparalogues per species	1519	*NA*	1543	*NA*	*NA*
	Average number of gene births per species	400	2805	593	*NA*	*NA*
	Number of 1:1 _(n=all)_ orthogroups	9538	6621	9595	5380	8643
	Number of 1:1 _(1<n<all)_ orthogroups	6711	10,231	6042	15,493	9756
Twenty genomes (Twelve FlyBase + eight modENCODE)	Average number of genes per species	15,055				
	Average number of orthologues per species	13,272	13,091	12,964	12,565	13,504
	Average number of inparalogues per species	1305	*NA*	1422	*NA*	*NA*
	Average number of gene births per species	468	2343	670	*NA*	*NA*
	Number of 1:1 _(n=all)_ orthogroups	6541	3912	7491	2555	5757
	Number of 1:1 _(1<n<all)_ orthogroups	14,047	16,681	11,880	25,694	16,799

Inparalogue predictions were carried out only in Orthonome and MultiMSOAR2. NA denotes the lack of inparalogue identification by OrthoDB and values that could not be calculated for MSOAR2 (since it has a different scoring method than Orthonome)

The greatly enhanced capability of Orthonome to resolve 1:many and many:many into 1:1 relationships was evident in an analysis of 11 gene families associated with stress tolerances and prone to rapid amplification and loss in insects [32]. Orthonome could resolve 45–133% more of these genes into 1:1 _(n=all)_ orthogroups than OrthoDB (Additional file 1: Table S3). For example, cytochrome P450 monooxygenases (P450s) were classified into 51 1:1 _(n=all)_ orthogroups by Orthonome compared to 22 by OrthoDB (Additional file 1: Table S3). An example of the improved orthologue assignment by Orthonome in a particular P450 gene cluster is illustrated in Fig. 3c.

### Detailed comparison of Orthonome with OrthoDB using the 20 genome data set

Compared to the 12 Flybase genomes above, the eight draft modENCODE genomes had significantly shorter post-assembly contig and scaffold N50 s (averages of 493Kb cf. 1.9 Mb and 1.1 Mb cf. 12.8 Mb respectively; Additional file 1: Table S1) and more missing members of the universal insect gene set identified by BUSCO (0.06% vs 1.65% respectively). Application of Orthonome to the eight draft modENCODE *Drosophila* genomes again achieved a further 0.5% improvement in orthologue recovery per species compared to OrthoDB. Compared to the 12 genomes analysis above, both pipelines had a reduction in the average number of orthologues recovery per species (OrthoDB: 1.8%; Orthonome:1.3%), which we can attribute to missing gene models in the additional draft genomes (Additional file 1: Fig. S1b). However, the number of 1:1 _(n=all)_ orthogroups per genome obtained from the combined data sets using Orthonome was 67% higher than the number obtained with OrthoDB, bearing out the value of Orthonome even for poorer quality genome assemblies.

The ability of Orthonome to identify orthologues and in particular to resolve 1:many and many:many orthogroups into 1:1 _(n=all)_ orthogroups with greater accuracy (Figs. 2 and 3c) also means that genes in each genome that do not fall into the latter class can be partitioned into three other classes, namely 1:1 _(1<n<all)_ orthogroups, inparalogues and *de novo* gene births (Table 1). OrthoDB does not provide for such partitioning. As noted earlier, another pipeline, OMA, which also produces orthogonal 1:1 prediction, does so with very low recall while also having a higher discordance compared to species phylogeny (Fig. 2).

The numbers of genes assigned to the orthogroup, inparalogue and *de novo* gene birth categories will be less prone to error in the 12 genome analysis than the 20 genome analyses because the 12 genome analysis gives the best estimate of the number of 1:1 _(n=all)_ orthogroups, as explained above. As such, the finding of an average of 1519 inparalogues above and 400 *de novo* gene births per genome in the 12 genome analysis (Table 1) represents the best estimates yet available for these two crucial sources of genetic novelty in the genus. Nevertheless we observed a negative relationship between the number of 1:1 _(n=all)_ orthogroups per genome and the quality of genome assembly/annotation (see above, Additional file 1: Note 2). As a result, although they are the most up-to-date at the time of writing, the estimates of gene birth and inparalogues are still very likely to be overestimates of the true values for these quantities because of the residual errors in the underlying assemblies and annotations.

## Conclusion

By comparing multiple pipelines we also find that Orthonome provides the best combination of accuracy and recall of orthologue assignments. Whereas previous pipelines are limited to classifying most genes in a genome into 1:many or many:many groups of orthologues, especially in draft genomes, Orthonome has separated the majority into 1:1 _(n=all)_ orthogroups (representing evolutionary conservation) or inparalogues and gene births (representing the genetic novelty from which new functions may evolve). While the current study is limited to Drosophila species, the application of Orthonome in other species orders ([32] and unpublished data) demonstrates a broader usability of the pipeline, though it may be possible to further improve the quality of orthologue prediction using substitution matrices optimised for specific phylogenetic lineages. As the quality of genome assemblies and annotations continues to improve, Orthonome and any subsequent improvements on it should provide increasingly accurate estimates of the various sources of genomic variation fundamental to evolutionary change.

## Additional file

Additional file 1: Supplementary notes, figures and tables. (DOCX 109 kb)

## Abbreviations

BL50: BLOSUM50 substitution matrix
BUSCO: Benchmarking Universal Single-Copy Orthologs
CSV: Comma separated values format
GFF3: General feature format version 3
HGT: Horizontal gene transfers
MCL: Markov cluster algorithm
OG: Orthogroup
OrthoXML: XML schema for storage of orthologue information
RSD: Reciprocal smallest distance
SOG: Super orthologue group
XLSX: Microsoft excel format
