# Supplementary material for: Orthonome – a new pipeline for predicting high quality orthologue gene sets applicable to complete and draft genomes
Source: BMC Genomics. 2017 Aug 31;18:673. doi: 10.1186/s12864-017-4079-6 (PMC5580312; doi:10.1186/s12864-017-4079-6)
Supplement: Additional file 1: — Supplementary notes, figures and tables. (DOCX 109 kb) [file 12864_2017_4079_MOESM1_ESM.docx]

**Supplementary Material**

**Supplementary notes**

**Supplementary Note 1: Correlation between genome quality and orthologue retrieval**

To evaluate the relationship between genome quality and orthologue retrieval by Orthonome, we calculated N50 statistics for the 12 well-curated Flybase and eight modENCODE draft *Drosophila* genomes (Table S1). When comparing the N50 statistic to the average number of 1:1 orthologues, the draft genomes clustered towards a lower intersect of the two axes as opposed to the 12 higher quality genomes (Figure S1a). We found a similar trend when comparing the number of annotated gene models with the average number of 1:1 orthologues (Figure S1b). The genome and annotation statistics were both positively correlated with the efficiency of orthologue retrieval in Orthonome (r = 0.58, P = 0.0075 & r = 0.67, P = 0.0012 respectively). We also found a significant negative correlation between genome N50 statistic and the number of gene births (r = -0.47, P = 0.034).

**Supplementary Note 2: Performance of other orthologue identification pipelines compared to Orthonome**

To ascertain the gain in orthologue identification capacity due to the additional features of the Orthonome pipeline, we utilised the peptide sequences used in Orthonome to identify orthologue clusters with OrthoDB, and the same input data for MSOAR2 as for Orthonome. For the twelve high quality FlyBase genomes we found that Orthonome was able to identify 9,538 1:1 _(n=all)_ orthogroups while OrthoDB and MultiMSOAR were able to identify 6,621 and 9,595 such orthogroups respectively (Tables 1 and S2).

To assess the effect of the lower quality of the draft genomes on orthologue recovery, we compared the analyses of all twenty genomes using Orthonome and OrthoDB. We found that the addition of the eight draft genomes significantly reduced the number of 1:1 _(n=all)_ orthogroups in both pipelines. We noted a 41% reduction in 1:1 _(n=all)_ orthogroups in OrthoDB (3,912, down from 6,621 for the twelve species analysis). Orthonome on the other hand produced 6,541 1:1 _(n=all)_ orthogroups, which is 31% down from the 9,538 orthogroups identified for the twelve high quality genomes (Table S2) but still 67% more than those predicted by OrthoDB. Therefore Orthonome appears more tolerant towards genome assembly and annotation errors that may have been introduced by draft genomes which have not been extensively curated like the twelve Flybase genomes.

When comparing the number of one-to-one orthologues identified in every pairwise comparison among species, we found an average increase of 2% (average of 195) in orthologue counts in Orthonome compared to MSOAR2. Since we observed a similar increase in orthologue count when comparing only the twelve high quality genomes or the eight draft genomes, we find that the usage of the *S’* scores compared to BLAST scores significantly increases the capacity of Orthonome to identify orthologous relationships.

We also noted an increase in orthologue counts by Orthonome compared to MSOAR2 in pairwise comparisons involving *D. melanogaster* despite it already being the best quality genome in our analysis (Table S4). Since the recovery of orthologues in a pairwise comparison could be negatively affected by fragmented genes, leading to lower BLAST scores, even comparisons between a high quality genome and a lower quality genome will result in a lower number of orthologues. The *S’* score calculates sequence similarity between genes by tolerating fragmentation of gene models, making it possible for Orthonome to capture a greater number of orthologues supported by phylogenetic and synteny measures.

One example of orthologue pairs only identified by Orthonome and missed by MSOAR2 as well as OrthoDB is the *D. melanogaster* gene FBgn0004554 and its *D. sechellia* counterpart. As shown in Figure 3A/B in the main text we found that most genes around FBgn0004554 had orthologues in *D. melanogaster* identified by all pipelines (black lines), barring two genes that are very small and present no sequence similarity to any other genes. FBgn0004554 on the other hand has an extremely low BLAST score with its syntenic counterpart in *D. sechellia* FBgn0170274 and aligned only the first 24 amino acids. However the S’ scoring system in Orthonome enabled it to correctly identify the two genes as orthologous to one another by calculating a higher score that takes into account the entire sequence length. This gene pair also satisfies the requirement of synteny and is therefore identified as a true orthologue.

**Supplementary Figures**


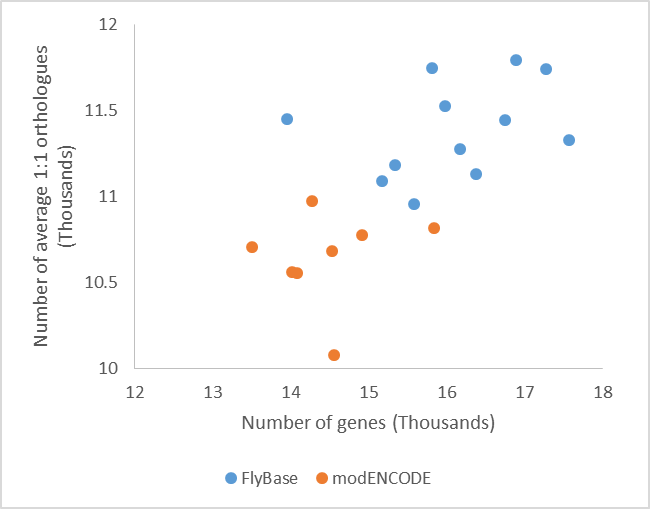

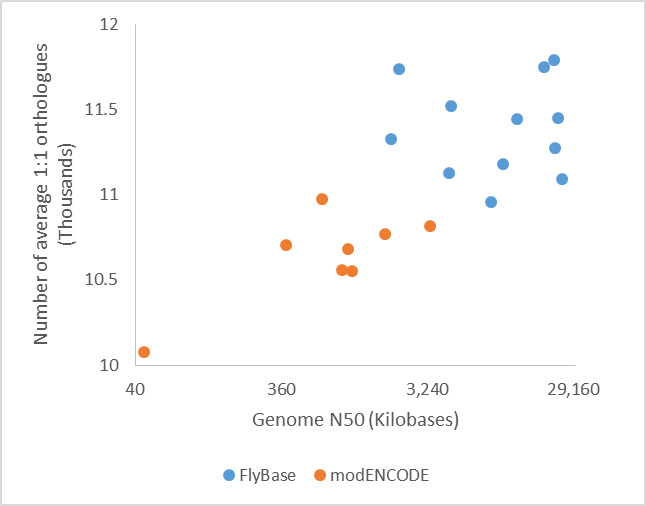


**Figure S1: Correlation between genome assembly and annotation quality and orthologue identification. (**a) N50 statistic vs. average number of 1:1 orthologues for each species. (b) Number of annotated gene models in a species with the average number of 1:1 orthologues for every species.

a

b

**Supplementary Tables
Supplementary Table 1:** Genome, orthologue and duplication statistics and data sources for all 20 Drosophila species analysed in the current study. These include the percentage of genes identified as orthologues by Orthonome and OrthoDB as well as genome statistics for each genome based on BUSCO analyses.

Additional file attached: Orthonome_SUPP_TableS1_SubmissionDraft.xlsx

**Supplementary Table 2:** Total number of 1:1 _(n=all)_ orthologroups identified by the Orthonome and OrthoDB pipelines in the 12 and 20 genome sets

| **Pipeline** | **12 Genome analysis** | **20 Genome analysis** | **% drop due to draft genomes** |
| --- | --- | --- | --- |
| Orthonome | 9538 | 6541 | 31 |
| OrthoDB | 6621 | 3912 | 41 |
| Improved performance of Orthonome | 44% | 67% |  |

**Supplementary Table 3:** Comparison between OrthoDB and Orthonome for number of orthogroups corresponding to eleven rapidly evolving gene families in *Drosophila* species

| **Gene Family** | ABCs | ACHRs | CCEs | GRs | GSTs | HSPs | MFS | OBPs | ORs | P450s | UGT |
| --- | --- | --- | --- | --- | --- | --- | --- | --- | --- | --- | --- |
| **Total genes in Drosophila melanogaster** | 56 | 12 | 23 | 60 | 34 | 9 | 66 | 52 | 61 | 87 | 17 |
| **Orthonome:** Number of unique orthogroups in | 55 | 12 | 23 | 57 | 33 | 9 | 65 | 51 | 58 | 77 | 16 |
| **OrthoDB:** Number of unique orthogroups in | 53 | 12 | 22 | 57 | 29 | 8 | 62 | 50 | 55 | 67 | 16 |
| **Orthonome:** Number of orthogroups with conserved 1-to-1 sets including all 12 species | 37 | 8 | 16 | 33 | 17 | 7 | 45 | 30 | 33 | 51 | 9 |
| **OrthoDB:** Number of orthogroups with conserved 1-to-1 sets including all 12 species | 24 | 5 | 11 | 17 | 9 | 3 | 28 | 20 | 13 | 22 | 5 |
| **Orthonome:** Improvement of orthogroup identification compared to OrthoDB (%) | 3.77 | 0.00 | 4.55 | 0.00 | 13.79 | 12.50 | 4.84 | 2.00 | 5.45 | 14.93 | 0.00 |
| **Orthonome**: Improvement of conserved 1-to-1 orthogroup identification compared to OrthoDB (%) | 54.17 | 60.00 | 45.45 | 94.12 | 88.89 | 133.33 | 60.71 | 50.00 | 153.85 | 131.82 | 80.00 |

**Families: ABC/ABC-like transporters (*ABCs*), acetyl-choline receptors (*ACHRs*), carboxyl/cholinesterases (*CCEs*), gustatory receptors (*GRs*), glutathione S-transferases (*GSTs*), Heatshock proteins (*HSPs*), Major facilitator superfamily of transporters (*MFS*), Odorant binding proteins (*OBPs*), odorant receptors (*ORs*), cytochrome P450s (*P450s*) and UDP-glucuronosyltransferase (*UGTs*).

**Supplementary Table 4:** Median and mean gain of orthologue pairs in Orthonome compared to MSOAR2 for the 20 Drosophila species

| **Species** | **Source** | **Median** | **Mean** | **Contig N50** | **BUSCO completion** |
| --- | --- | --- | --- | --- | --- |
| Drosophila ananassae | Flybase | 172 | 186.47 | 99399 | 98% |
| Drosophila erecta | Flybase | 142 | 170.26 | 455916 | 99% |
| Drosophila grimshawi | Flybase | 180 | 192.21 | 92106 | 99% |
| Drosophila melanogaster | Flybase | 113 | 143.53 | 21485538 | 98% |
| Drosophila mojavensis | Flybase | 202 | 218.63 | 124510 | 98% |
| Drosophila persimilis | Flybase | 219 | 232.63 | 20311 | 93% |
| Drosophila pseudoobscura | Flybase | 607 | 588.16 | 203957 | 93% |
| Drosophila sechellia | Flybase | 118 | 168.53 | 42955 | 96% |
| Drosophila simulans | Flybase | 166 | 201.21 | 15927 | 84% |
| Drosophila virilis | Flybase | 182 | 210.53 | 123628 | 99% |
| Drosophila willistoni | Flybase | 169 | 197.26 | 183168 | 99% |
| Drosophila yakuba | Flybase | 112 | 22.63 | 255016 | 98% |
| Drosophila biarmipes | modENCODE | 182 | 196.05 | 149400 | 96% |
| Drosophila bipectinata | modENCODE | 201 | 84.32 | 467944 | 97% |
| Drosophila elegans | modENCODE | 188 | 200.26 | 937343 | 96% |
| Drosophila eugracilis | modENCODE | 162 | 173.05 | 658447 | 95% |
| Drosophila ficusphila | modENCODE | 161 | 183.21 | 829588 | 95% |
| Drosophila kikkawai | modENCODE | 159 | 168.32 | 575074 | 96% |
| Drosophila takahashii | modENCODE | 175 | 183.42 | 298321 | 94% |
| Drosophila rhopaloa | modENCODE | 172 | 184.79 | 35496 | 93% |
